# Effects of Dietary Supplementation of Bile Acids on Growth, Glucose Metabolism, and Intestinal Health of Spotted Seabass (*Lateolabrax maculatus*)

**DOI:** 10.3390/ani14091299

**Published:** 2024-04-25

**Authors:** Yongping Liu, Xiao Li, Jibin Lin, Kai Song, Xueshan Li, Ling Wang, Chunxiao Zhang, Kangle Lu

**Affiliations:** 1 State Key Laboratory for Mariculture Breeding, Fisheries College of Jimei University, Xiamen 361021, China; yupp627315@163.com (Y.L.); xiaolee@jmu.edu.cn (X.L.);; 2 Xiamen Key Laboratory for Feed Quality Testing and Safety Evaluation, Fisheries College of Jimei University, Xiamen 361021, China; 3 Key Laboratory of Healthy Mariculture for the East China Sea, Ministry of Agriculture and Rural Affairs, Jimei University, Xiamen 361021, China

**Keywords:** bile acid, *Lateolabrax maculatus*, growth, glucose metabolism, intestinal health

## Abstract

**Simple Summary:**

Bile acids are synthesized from cholesterol in the liver and play a crucial role in the metabolism of dietary lipids as emulsifiers. Exogenous bile acids supplementation can promote the growth of fish reared at optimal water temperatures by increasing feed intake and digestion, enhancing antioxidant capacity, and improving gut health. However, whether they also have positive effects on fish reared at high water temperatures remains unclear. In this study, we investigated the effects of dietary bile acids on growth, glucose metabolism, and intestinal health in spotted seabass reared at high temperatures (33 °C). The results demonstrated that dietary bile acids promoted fish growth, improved glucose metabolism, and maintained intestinal health in spotted seabass.

**Abstract:**

An 8-week feeding trial was performed to investigate the effects of dietary bile acids on growth, glucose metabolism, and intestinal health in spotted seabass (*Lateolabrax maculatus*) reared at high temperatures (33 °C). The fish (20.09 ± 1.12 g) were fed diets supplemented with bile acids: 0 (Con), 400 (BA400), 800 (BA800), and 1200 (BA1200) mg/kg, respectively. The results showed that the growth was promoted in fish at the BA800 treatment compared with the control (*p* < 0.05). Increased enzyme activities and transcripts of gluconeogenesis in the liver were observed, whereas decreased enzyme activities and transcripts of glycolysis, as well as glycogen content, were shown in the BA800 treatment (*p* < 0.05). The transcripts of bile acid receptors *fxr* in the liver were up-regulated in the BA800 treatment (*p* < 0.05). A bile acid supplementation of 800 mg/kg improved the morphological structure in the intestine. Meanwhile, intestinal antioxidant physiology and activities of lipase and trypsin were enhanced in the BA800 treatment. The transcripts of genes and immunofluorescence intensity related to pro-inflammation cytokines (*il-1β*, *il-8*, and *tnf-α*) were inhibited, while those of genes related to anti-inflammation (*il-10* and *tgf-β*) were induced in the BA800 treatment. Furthermore, transcripts of genes related to the NF-κB pathway in the intestine (*nfκb*, *ikkα*, *ikkβ*, and *ikbα1*) were down-regulated in the BA800 treatment. This study demonstrates that a dietary bile acid supplementation of 800 mg/kg could promote growth, improve glucose metabolism in the liver, and enhance intestinal health by increasing digestive enzyme activity and antioxidant capacity and inhibiting inflammatory response in *L. maculatus*.

## 1. Introduction

Spotted seabass (*Lateolabrax maculatus*) has become a major fish cultured in southern China due to its high nutritional and economic value [1]. Its total production reached approximately 218,000 tons in 2022 [2]. The optimal water temperature range for *L. maculatus* is from 16 °C to 28 °C [3]. In the main farming areas for *L. maculatus*, however, water temperature often reached 33 °C or higher along the southeast coast of China in summer. Water temperature is one of the most pervasive and influential environmental factors in intensive aquaculture, which directly impacts almost all aspects of fish physiology [4,5]. A high water temperature may lead to an excessive accumulation of reactive oxygen species (ROS) [6,7], causing energy metabolism disorders in organisms [8,9], ultimately resulting in various adverse effects on growth, antioxidant capacity, immunity response, and survival in fish [10,11,12]. Our recent studies demonstrated that a high water temperature of 33 °C may lead to a decrease in growth [1], antioxidant capacity, immune response, and the survival of *L. maculatus*, causing hepatic metabolic disorders [13] and intestinal mucosal damage [14] in *L. maculatus*. Thus, the negative effects of high temperatures on fish have become one of the main obstacles to the development of the aquaculture industry [15].

Bile acid is synthesized by cholesterol in the liver and is mainly stored in the gallbladder as bile salts [16]. In general, bile acids primarily enhance the efficiency of dietary lipid digestion in fish by acting as emulsifiers and also play a crucial role in activating lipases [17,18] and improving the absorption and transportation of lipid and lipid-soluble nutrients in fish [19]. Meanwhile, as a signaling molecule, bile acids may be involved in regulating the metabolism of lipids and glucose in organisms [20]. Furthermore, further research indicated that dietary supplementation of bile acids can also promote antioxidant capacity [21], improve anti-inflammatory properties [22], enhance immune response [23], and maintain intestinal health [24] in fish.

Given the importance of bile acids in the physiological metabolism of the body, their multiple biological properties for fish are also increasingly recognized [25,26,27]. Surprisingly, a previous study in turbot (*Scophthalmus maximus*) has found that high water temperature inhibited the expression of the *cyp27a1* gene in bile acid synthesis compared with the optimal water temperature [28]. In agreement with this, the data of bile acid metabolomics from our study indicated that the synthesis of endogenous bile acid in *L. maculatus* reared at a high water temperature (33 °C) was significantly lower (*p* < 0.05) than that of fish reared at the optimal temperature (27 °C) (See Figure S1). However, it remains largely unclear whether bile acid supplementation can mitigate the negative effects of high water temperature on fish. We tested the hypothesis that the supplementation of appropriate levels of exogenous bile acids could be beneficial in mitigating the negative effects on growth and physiological homeostasis caused by high temperatures on fish. Thus, the main objective of this study was to evaluate the effects of dietary supplementation of bile acids on growth performance, glucose metabolism, and intestinal health in *L. maculatus* reared under high water-temperature conditions (33 °C).

## 2. Materials and Methods

### 2.1. Ethics Statement

All the animal trials in this study were conducted according to the guidelines of the Ethics Committee of Care and Use for Laboratory Animals of Jimei University, Xiamen, Fujian, China (No. JMU202303004, Approval date: 10 March 2023).

### 2.2. Preparation of Experimental Diets

Four isonitrogenous (43.0% crude protein) and isolipidic (12% crude lipid) diets were prepared, four diets of which were supplemented with different levels of bile acids mixture (0, 400, 800, and 1200 mg/kg). These treatments were defined as Con, BA400, BA800, and BA1200, respectively. The bile acid mixture (including 75.2% hyodeoxycholic acid (HDCA), 17.4% chenodeoxycholic acid (CDCA), and 4.2% hyodeoxycholicacid (HCA)) was observed from Longchang Animal Health Co., Ltd. (Dezhou, Shandong, China). In this study, fishmeal, soybean meal, and poultry by-product meal were used as the main protein sources, and fish oil and soybean oil were used as lipid sources. The formulation and proximate composition of five diets for juvenile *L. maculatus* are shown in Table 1. The diets were prepared as described in our previous study [1]. Prior to diet preparation, all ingredients were crushed and sieved below 60-mesh. After mixing all the dry ingredients, the oil mixture was added, followed by the addition of 30% (*w*/*w*) deionized distilled water to form a dough. The feed at ~2.5 mm diameter was made using a cold-extruded pellet producer. The feeds were dried at 50 °C for 10 h, then stored at −20 °C until use.

### 2.3. Experimental Design and Feeding Trial

One thousand juvenile *L. maculatus* were obtained from the commercial fish breeding farm (Zhangzhou, Fujian, China) and transferred to the aquaculture research facilities of Jimei University. The fish were housed in a 1000 L semi-static system with a pre-set temperature of 33 °C. They were fed the same control diets twice daily for 2 weeks to acclimate to the experimental environment. After acclimation, 240 fish (20.09 ± 1.12 g) were randomly grouped into twelve 150 L tanks (20 fish per tank) connected to a recirculating aquaculture system with a pre-set temperature of 33 °C. Three replicates were allocated to each dietary bile acid treatment. During the feeding trial for 8 weeks, fish were fed the bile acid-supplemented diet at ~3% of their weight twice daily at 8:00 and 17:00. Uneaten food and feces were removed after 30 min of feeding. The dechlorinated tap water was renewed, and feed intake was recorded daily. The conditions of the culture were as follows: water temperature at 33 ± 0.5 °C, 12 h light–12 h dark photoperiod, total ammonia–nitrogen < 0.35 mg/L, dissolved oxygen ≥ 6.2 mg/L, and pH 7.0~7.4.

### 2.4. Sample Collection

At the end of the feeding experiment, the experimental fish were starved for 24 h to allow emptying of the intestine. All fish from each tank were anesthetized with eugenol (0.1 mL/L) for ~1 min to record the body length and weight and for subsequent sample collection. Two fish were pooled as one composite sample randomly selected from each tank and stored at −20 °C to determine the proximate composition of whole body (*n* = 3). The livers of 2 fish were pooled as one composite sample per tank (*n* = 3) was collected for measuring the enzyme activity related to glucose metabolism. The livers of 2 fish were pooled as one composite sample per tank was collected for measuring the transcripts of genes related to bile acid receptors and glucose metabolism (*n* = 3). The midgut of 2 fish were pooled as one composite sample per tank was collected for measuring the antioxidant capacity and digestive enzyme activity (*n* = 3). The midgut of 2 fish were pooled as one composite sample per tank was collected for measuring the transcripts of genes related to inflammatory cytokines and NF-κB pathway (*n* = 3). The midgut of 2 fish per tank was fixed in a 4% paraformaldehyde solution (Biosharp, Beijing, China) for histological examinations and immunofluorescence detection (*n* = 6).

### 2.5. Proximate Composition of Diets and Whole Fish

The proximate compositions of diets and whole-body were measured according to the standard procedures [29]. Crude protein content was measured using a Kjeldahl System (2300-Auto-analyzer, FOSS, Hillerød, Denmark). Crude lipid content was analyzed following Soxhlet’s extraction method. For the determination of moisture content, samples were dried to a constant weight at 105 °C. Ash content was measured by combusting using a muffle furnace at 550 °C for 8 h.

### 2.6. Glucose Metabolism of Liver

The liver weighing 0.1 g from each replicate was weighed, homogenized at 4 °C using a 9-fold volume-to-weight ratio of phosphate-buffered saline (PBS, pH = 7.4), and centrifuged at 2500 rpm for 10 min. The supernatants were collected and allocated for the subsequent assays. Enzyme activities of hexokinase (HK, No. A077-3-1), pyruvate kinase (PK, No. A076-1-1), phosphofructokinase (PFK, No. A129-1-1), phosphoenolpyruvate carboxykinase (PEPCK, No. A131-1-1), and phosphoenolpyruvate carboxylase (PEPC, No. A130-1-1) in the liver samples were determined following the protocols of commercial assay kits from Jiancheng Bioengineering Institute (Nanjing, China). Results were obtained based on the wet weight of the samples.

The liver glycogen content was measured in strict accordance with the instructions provided by Nanjing Jiancheng Bioengineering Institute (No. A043-1-1). In brief, 95 mg of liver samples were hydrolyzed in a boiling water bath for 20 min with an acidic solution (1:3 weight to volume ratio). The resulting hydrolysate was diluted with deionized water to prepare the glycogen assay solution. A 0.1 mL aliquot of this solution was mixed with 0.9 mL deionized water, and a chromogenic agent was heated for 5 min. OD values were then measured at 620 nm after cooling.

### 2.7. Intestinal Antioxidant Capacity and Digestive Enzyme Activity

The mid-intestine weighing 0.1 g from each replicate was weighed, homogenized at 4 °C using a 9-fold volume-to-weight ratio of phosphate-buffered saline (PBS, pH = 7.4), and centrifuged at 2500 rpm for 10 min. The activities of three digestive enzymes (lipase, trypsin, and amylase) as well as total antioxidant capacity (T-AOC, No. A015-2-1), superoxide dismutase (SOD, No. A001-3-2) activity, and malondialdehyde (MDA, No. A003-1-2) content in the intestine of experimental fish were determined according to the methods described in the commercial assay kits (Jiancheng Bioengineering Institute, Nanjing, China).

### 2.8. Histological Analyses and Immunofluorescence Detection in the Intestine

Histological analyses of the mid-intestine samples were performed following the method with minor modifications [30]. In brief, paraformaldehyde-fixed intestines were cut off with a length of ~0.6 cm, followed by dehydration in graded alcohols and clearance in xylene. Afterward, the muscle samples were embedded in paraffin wax, sectioned at 6 μm thickness, and stained with hematoxylin and eosin (H&E). Six randomly chosen fields of the intestine from each treatment were observed under a microscope (Leica DM5500B, Heidelberg, Baden-Württemberg, Germany). The villus length (VL), villus width (VW), and thicknesses of muscularis (MT) were determined using the Image-Pro Plus 0.53 software connected to the microscope (*n* = 6).

The procedures of immunofluorescence staining for inflammatory cytokines (IL-1β, IL-8, TNF-α) were carried out consistently with H&E staining prior to embedding. Subsequently, antigen retrieval was performed, followed by encircling sealing serum, addition of primary and secondary antibodies, staining of cell nuclei with DAPI staining solution, tissue staining with autofluorescence quencher B solution, and sealing with anti-fluorescence quenching sealer before being placed under an orthogonal fluorescence microscope (Nikon, NIKON ECLIPSE C1, Tokyo, Tokyo Metropolis, Japan) for observation [31].

### 2.9. RNA Extraction and Quantitative Real-Time PCR (qPCR) Assay

The total RNA extraction, RNA quantity and quality, synthesis cDNA, and qPCR procedures were conducted following the previously described method with modifications [30]. Extraction of total RNA from the liver and intestine was performed according to the instructions of FastPure Cell/Tissue Total RNA Isolation Kit (Vazyme Biotechnology Co., Ltd., Nanjing, China). The concentration and purity of extracted total RNA were quantified using a spectrophotometer (NanoDrop Technologies, Waltham, MA, USA) at a wavelength of 260/280 nm, and then the integrity of the RNA was assessed through 1.5% agarose gel electrophoresis. For each sample, 3 μg (0.15 μg/μL) RNA was reverse-transcribed into cDNA for quantitative real-time PCR (RT-qPCR) following the protocol of commercial kit (R211-01, Vazyme, Nanjing, Jiangsu, China). The transcriptional expression of genes in the liver and intestine was quantified by real-time quantitative PCR (QuantStudio™ 6 Flex, AppliedBiosystems, Waltham, MA, USA). In brief, the RT-qPCR program was run at 95 °C for 30 s, followed by 40 cycles of 95 °C for 15 s and 60 °C for 30 s. After the PCR reaction, melting curve analysis was performed to confirm the specificity of the genes.

In this study, the sequences of the primers were designed using Primer 5.0 software and synthesized by Genewiz Co., Ltd., Suzhou, China (Table 2). The relative expression of target genes was normalized to β-actin and 18s using the 2^−ΔΔCt^ method [32]. In addition, the amplification efficiency of all primers in this study was verified between 90 and 110%.

### 2.10. Statistical Analyses

All data were analyzed following one-way analysis of variance (ANOVA) using SPSS 22.0 statistical software. Prior to statistical analysis, Kolmogorov−Smirnov and Levene’s tests were used to test the assumptions of normality and homogeneity of variance, respectively. Multiple comparisons were performed using Tukey’s test to analyze the differences between the experimental groups. The level of significance was set at *p* < 0.05. All results are presented as mean ± standard error of the mean (SE).

## 3. Results

### 3.1. Growth Performance and Proximate Composition Analyses

After an 8-week feeding trial, the final body weight (FBW) and weight gain (WG) of *L. maculatus* were significantly increased in the BA800 treatment compared to the control fish (Table 3) (*p* < 0.05). Meanwhile, the feed intake (FI) was elevated in the BA400, BA800, and BA1200 treatments (*p* < 0.05). However, no significant differences in the feed conversion rate (FCR), abdominal fat ratio (AFR), or survival of *L. maculatus* were observed among all treatments.

The results of proximate composition analyses in the whole fish were reported in Table 4. In comparison to the control treatment, an increased content of crude lipid was observed in the BA800 treatment (*p* < 0.05), while an opposite result was shown in crude ash content (*p* < 0.05). Furthermore, there were no significant differences in the moisture and crude protein content of *L. maculatus* among all treatments (*p* > 0.05).

### 3.2. Glucose Metabolism of Liver

**Figure 1:**
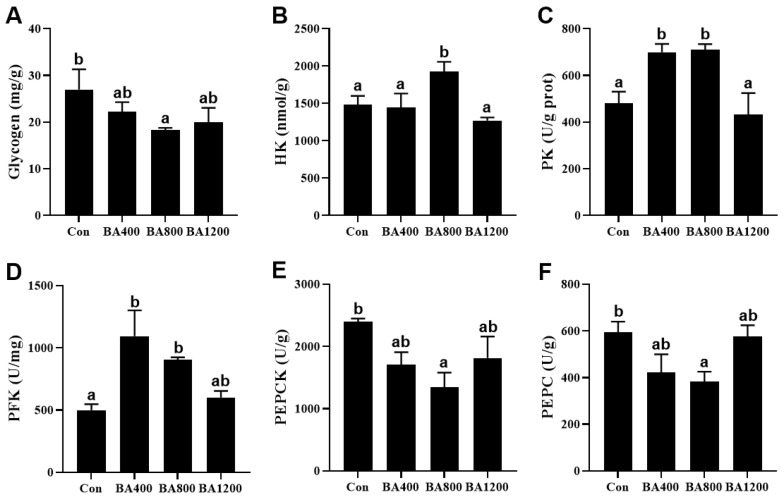
Effects of dietary bile acid on the glycogen content (panel (**A**)) and enzyme activities of hexokinase (panel (**B**), HK), pyruvate kinase (panel (**C**), PK), phosphofructokinase (panel (**D**), PFK), phosphoenolpyruvate carboxykinase (panel (**E**), PEPCK), phosphoenolpyruvate carboxylase (panel (**F**), PEPC) in the liver of *L. maculatus*. Bars with different letters are different at *p* < 0.05.

As shown in Figure 1, compared to the control treatment, glycogen content and activities of phosphoenolpyruvate carboxykinase (PEPCK) and phosphoenolpyruvate carboxylase (PEPC) in the liver of *L. maculatus* were significantly reduced (*p* < 0.05) in the BA800 treatment, whereas there was no difference among the other treatments (*p* > 0.05). Activities of hexokinase (HK) in the liver of fish were increased (*p* < 0.05) in the BA800 treatment. Meanwhile, activities of pyruvate kinase (PK) and phosphofructokinase (PFK) in the liver of fish were increased (*p* < 0.05) in the BA400 and BA800 treatments but decreased at the BA1200 treatment. The maximum values of these three parameters were obtained in the BA800, BA800, and BA400 treatments, respectively.

**Figure 2:**
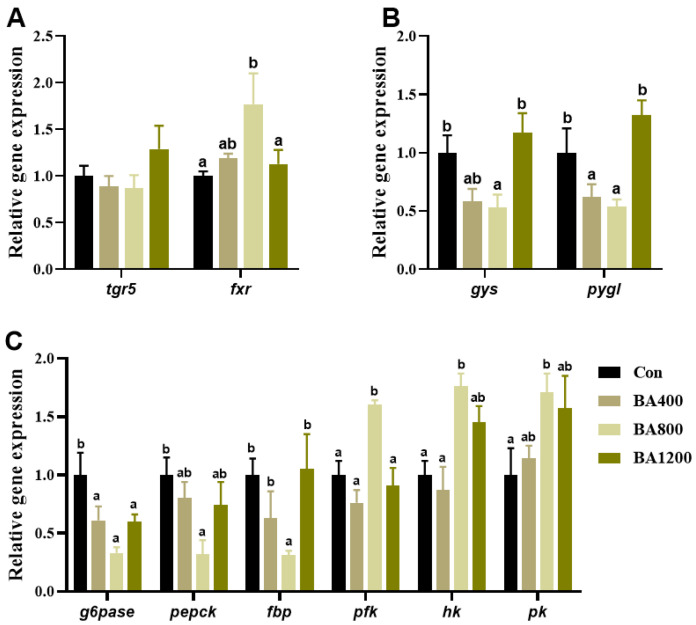
Effects of dietary bile acids on the transcriptional expression of genes related to bile acid receptors (panel (**A**)), glycogen synthesis (*gys*), glycogen catabolism (*pygl*) (panel (**B**)), and glycolysis (*g6pase*, *pepck*, and *fbp*) and gluconeogenesis (*pfk*, *hk*, and *pk*) (panel (**C**)) in the liver of *L. maculatus*. Bars with different letters are different at *p* < 0.05.

The transcripts of the farnesoid X receptor (*fxr*) related to bile acid receptors in the liver from the BA800 treatment were significantly up-regulated (*p* < 0.05) compared to the control treatment, whereas the transcripts of trans-membrane G protein-coupled receptor-5 (*tgr5*) were not affected by the addition of dietary bile acids (*p* > 0.05) (Figure 2A). Transcripts of glycogen synthase (*gys*) and phosphorylase glycogen L (*pygl*) in the liver displayed an initial down-regulation followed by an up-regulation with the increase in dietary bile acid levels and were significantly lower in the BA800 treatment compared to the other treatments (*p* < 0.05) (Figure 2B). Meanwhile, the transcripts of glucose-6-phosphatase (*g6pase*) in the liver were inhibited by dietary bile acid, whereas transcripts of *pepck* and fructose 1,6-bisphosphatase (*fbp*) were only inhibited in the BA800 treatment (*p* < 0.05), with no alteration among the other treatments (*p* > 0.05) (Figure 2C). Furthermore, transcripts of *pfk, hk*, and *pk* in the liver were significantly up-regulated in the BA800 treatment compared to the control treatment (*p* < 0.05), while no differences were found among the other treatments (*p* > 0.05) (Figure 2C).

### 3.3. Histological and Morphological Analyses in the Intestine

**Figure 3:**
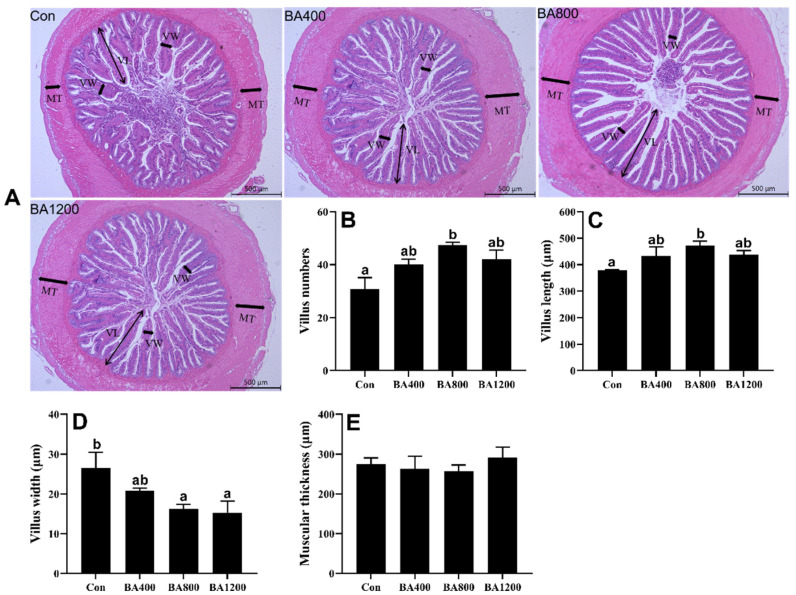
Representative images of intestinal morphology (bar: 500 μm) in *L. maculatus* fed different levels of bile acids for 8 weeks (panel (**A**)) and morphological alterations (panels (**B**–**E**)). VL: villus length; VW: villus width; MT: muscular thickness (*n* = 3). Bars with different letters are different at *p* < 0.05.

The histological analysis showed that control fish showed a typical inflammatory response in the intestine, including irregular arrangement of intestinal mucosa epithelium (Figure 3A). However, the damage to the morphology of the intestinal villus was significantly improved with the supplementation of dietary bile acids. The number and length of villus exhibited an increasing trend with increasing the levels of dietary bile acid supplementation, with both being significantly higher in the BA800 treatment compared to control fish (Figure 3B,C) (*p* < 0.05). Meanwhile, the width of the villus exhibited an opposite trend, and a significant decrease in villus width was observed in the BA400 and BA800 treatments compared to control fish (Figure 3D) (*p* < 0.05). In addition, the muscular thickness of the intestine was not altered by dietary bile acid supplementation.

### 3.4. Antioxidant Capacity in the Intestine

**Figure 4:**
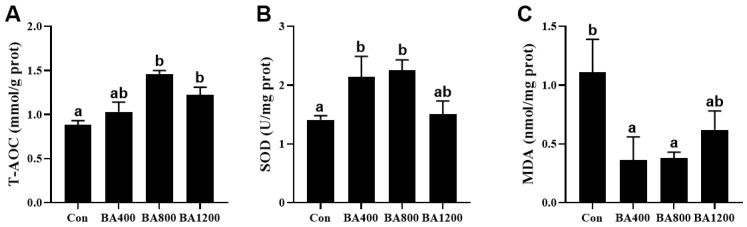
Effects of dietary bile acids on the total antioxidant capacity (T-AOC, panel (**A**)) and activity of superoxide dismutase (SOD, panel (**B**)) and malondialdehyde (MDA, panel (**C**)) in the intestine of *L. maculatus*. Bars with different letters are different at *p* < 0.05.

The antioxidant capacity in the intestine of *L. maculatus* was elevated by increasing the levels of dietary bile acids supplementation. Compared to the control treatment, total antioxidant capacity (T-AOC) was increased in the BA800 and BA1200 treatments (Figure 4A), while the activity of superoxide dismutase (SOD) was significantly induced by the BA400 and BA800 treatments (Figure 4B) (*p* < 0.05). Meanwhile, with the elevation of bile acid levels, MDA content in the BA400 and BA800 treatments exhibited a significant reduction compared to the control (Figure 4C) (*p* < 0.05).

### 3.5. Digestive Enzyme Activities in the Intestine

**Figure 5:**
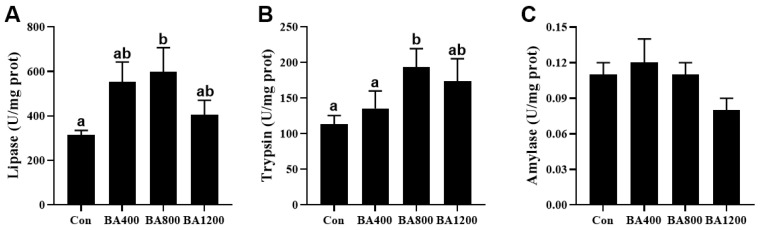
Effects of dietary bile acids on the activities of lipase (panel (**A**)), trypsin (panel (**B**)), and amylase (panel (**C**)) in the intestine of *L. maculatus*. Bars with different letters are different at *p* < 0.05.

The activity of digestive enzymes in the intestine of *L. maculatus* was altered by increasing the levels of dietary bile acid supplementation. The activities of lipase and trypsin exhibited an initial increase followed by a subsequent decrease, with their highest value observed in the BA800 treatment (Figure 5A) (*p* < 0.05). The activity of amylase was no different among the dietary bile acid supplementation treatments (Figure 5C) (*p* > 0.05).

### 3.6. Immunofluorescence and Transcriptional Expression of Genes Related to Inflammatory Cytokines

**Figure 6:**
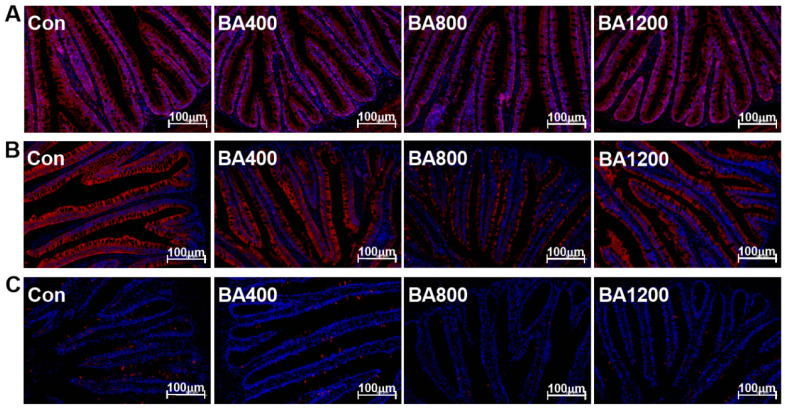
Representative immunofluorescence images of inflammatory cytokines in the intestine of *L. maculatus* fed different levels of bile acids for 60 d (bar: 100 μm). (**A**) IL-1β, interleukin-1β; (**B**) IL-8, interleukin-8; (**C**) TNF-α, tumor necrosis factor-α (*n* = 3). The nucleus is stained blue, and the target inflammatory cytokines are stained red in the image above.

**Figure 7:**
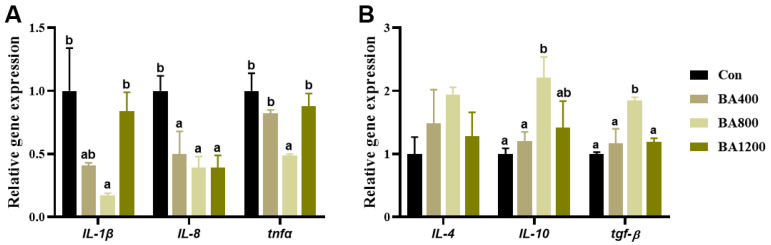
Effects of dietary bile acids on the transcriptional expression of genes related to pro-inflammatory (panel (**A**)) and anti-inflammatory cytokines (panel (**B**)) in the intestine of *L. maculatus*. Bars with different letters are different at *p* < 0.05.

The immunofluorescence and transcriptional expression of genes related to inflammatory cytokines in the intestine are shown in Figure 6 and Figure 7. The fluorescence intensities of intestinal pro-inflammatory cytokines interleukin-1β (IL-1β), interleukin-8 (IL-8), and tumor necrosis factor-α (TNF-α) in the BA800 treatment were significantly lower than in the other treatments (Figure 6) (*p* < 0.05). Similarly, transcripts of *il-1β*, *il-8*, and *tnf-α* were down-regulated as bile acid levels increased up to the BA800 diet and then up-regulated (Figure 7A) (*p* < 0.05). Meanwhile, transcripts of intestinal anti-inflammatory cytokines *il-10* and transforming growth factor-β (*tgf-β*) were significantly up-regulated as bile acid levels increased up to the BA800 diet, followed by down-regulation (Figure 7B) (*p* < 0.05). However, the transcriptional expression of *il-4* was no different among the dietary bile acid supplementation treatments (*p* > 0.05).

### 3.7. Transcriptional Expression of Genes Related to NF-κB Pathway

**Figure 8:**
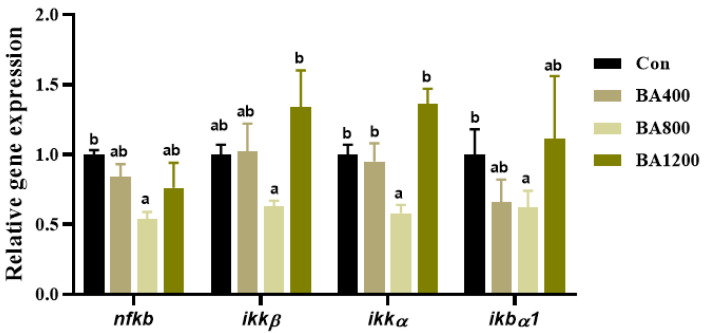
Effects of dietary bile acids on the transcriptional expression of genes related to nuclear factor κB (NF-κB) pathway in the intestine of *L. maculatus*. Bars with different letters are different at *p* < 0.05.

As shown in Figure 8, transcripts of nuclear factor κB (*nf-κb*), an inhibitor of κB kinase-α (*ikkα*), and NF-κB inhibitory protein α1 (*ikbα1*) in the intestine of *L. maculatus* were significantly down-regulated as bile acid levels increased up to BA800 diet, followed by up-regulation (Figure 8) (*p* < 0.05). For the inhibitor of κB kinase-α (*ikkα*), compared to the control, there was no difference in the transcripts across all dietary bile acid supplementation treatments, but the transcript of *ikkα* in the BA800 treatment was lower than that in the BA1200 treatment.

## 4. Discussion

As indicated by many previous studies [14,33,34], three biological replicates were set up for each treatment in this study, and the samples from two fish were mixed together as one replicate to reduce variability between individual samples. Due to limitations in sample size and difficulties in obtaining certain samples (i.e., serum), such experimental methods are often used in research on aquatic animals [30]. In this study, the FBW and WG of *L. maculatus* were promoted by supplementing 800 mg/kg of bile acids in the diet, suggesting that the appropriate level of exogenous bile acid supplementation in the diet can promote the growth of *L. maculatus*. Similarly, the promoting effect of appropriate supplementation levels of dietary bile acids on the growth of fish has been widely reported in tilapia (*Oreochromis niloticus*) [35], largemouth bass (*Micropterus salmoides*) [36], European eel (*Anguilla anguilla*) [37], grass carp (*Ctenopharyngodon idella*) [27], turbot (*Scophthalmus maximus*) [38]. Meanwhile, the FI of *L. maculatus* increased with the increase in dietary bile acid levels, which seemed to be attributed to the promotion of growth. Previous studies have also indicated that the promotion of fish growth by bile acid is often positively correlated with increased FI [39], which is an undisputed result. Bile acids play a crucial role in regulating lipid metabolism by effectively enhancing the emulsification and transportation of lipids [40], as evidenced by the slight increase in abdominal fat ratio and significant accumulation in crude lipids in the whole fish with dietary supplementation of bile acids up to 800 mg/kg in this study. Furthermore, increased activity of intestinal lipase in fish fed with the diet supplemented with 800 mg/kg of bile acids also further demonstrates the beneficial effect of bile acids on lipid absorption. More similar results on the promotion of lipid digestion and absorption by the supplementation of exogenous bile acids have been obtained in large yellow croaker [33], largemouth bass [41], tilapia (*Oreochromis niloticus*) [26], and rainbow trout (*Oncorhynchus mykiss*) [42].

The liver is an important tissue that regulates glucose metabolism in organisms [43]. TGR5 and FXR are the two most important receptors for bile acids in animals, playing a crucial role in the molecular pathways regulating glucose metabolism involving bile acids [44,45]. It has been reported that FXR, as a nuclear transcription factor, regulates glucose and lipid metabolism balance through pyruvate dehydrogenase kinase 4 [46]. In this study, the up-regulated transcript of *fxr* was found in the liver of fish fed with a diet supplemented with 800 mg/kg of bile acids. Meanwhile, the same dietary bile acids level down-regulated transcripts of the genes related to gluconeogenesis (*g6pase*, *pepck*, and *fbp*) and up-regulated the genes related to glycolysis (*pfk*, *hk*, and *pk*). These findings are consistent with the effect of 800 mg/kg dietary bile acids on the activity of enzymes related to glucose metabolism in the liver. A previous study has also shown that bile acids and FXR inhibit the activities of PEPCK, G6Pase, and FBPase, all of which are enzymes involved in the hepatic gluconeogenesis pathway [47]. In addition, in this study, the reduction of hepatic glycogen content at the level of 800 mg/kg dietary bile acids further strengthens our evidence, suggesting that dietary bile acid supplementation promotes the conversion of glycogen to glucose for energy supply, thereby reducing the accumulation of glycogen in the liver of *L. maculatus* reared at high water temperature. Overall, these previous studies, together with our own results, confirm that the supplementing exogenous bile acids may inhibit hepatic gluconeogenesis and promote hepatic glycolysis by activating the bile acid receptor FXR, regulating glucose metabolism in fish [44].

Intestinal health is crucial for maintaining the normal growth and survival of fish [48]. The intestine in fish serves as the primary site for the digestion of food and absorption of nutrients, relying on the microvilli at the edges of the single-layer columnar epithelial cells [30]. Once intestinal mucosa is impaired, it can lead to the malabsorption of nutrients, increased membrane permeability, and inflammatory infiltration, thereby causing a potential risk to the growth and health of fish [49]. In the present study, the control fish exhibited a typical intestinal inflammation accompanied by a short and irregular arrangement of intestinal mucosa epithelium. However, dietary bile acid supplementation of 800 mg/kg increased the number and length of villus and decreased the villus width. Meanwhile, in this study, dietary bile acid supplementation of 800 mg/kg down-regulated the transcripts and fluorescence intensities of pro-inflammatory cytokines (*il-1β*, *il-8*, and *tnf-α*) and up-regulated the transcripts of anti-inflammatory cytokines (*il-10* and *tgf-β*) in the intestine. These findings are consistent with a previous study showing that dietary bile acid supplementation inhibits the transcripts of *il-1β*, *il-8*, and *tnf-α* in the intestine by activating intestinal bile acid reporter TGR5 in grass carp [27]. NF-κB is one of the key nuclear transcription factors involved in inflammatory responses in organisms, regulating the transcription of many genes related to inflammation [50]. Previous studies have demonstrated that bile acids inhibit the nuclear translocation of *nfκb* to down-regulate the transcription of *nfκb*-dependent pro-inflammatory cytokines (i.e., *il-1β*, *il-8*, and *tnf-α*), thereby achieving the goal of inhibiting intestinal inflammation [27]. In the present study, dietary bile acid supplementation of 800 mg/kg down-regulated the transcripts of *nfκb* and its downstream regulation of key genes (*ikkα*, *ikkβ*, and *ikbα1*). Overall, synthesizing our previous research, it is clear that thermal stress-induced intestinal inflammation can cause damage to the intestinal mucosa in *L. maculatus* [14]. Nevertheless, optimal exogenous bile acid supplementation in diets has been shown to effectively mitigate this negative impact, protect intestinal barrier integrity, and promote intestinal health in *L. maculatus*.

Intestinal digestive enzymes also play a crucial role in the digestion and absorption of nutrients [51]. In this study, the increased activity of lipase can be recognized as an indicator that lipid utilization is promoted, which has been discussed in the preceding text. Meanwhile, dietary bile acid supplementation of 800 mg/kg increased trypsin activity in the intestine of *L. maculatus* and may imply an overall promotional effect of bile acids on the digestion and absorption of nutrients in feed rather than just the utilization of lipids. A similar result has also been reported that trypsin activity was induced when dietary bile acid was given at 130 mg/kg in thinlip mullet [39].

Antioxidant capacity is a crucial index for evaluating the health and oxidative stress status of fish [52]. Generally, oxidative stress occurs due to an imbalance between the generation and elimination of ROS in organisms [53]. The accumulation of undetoxified ROS can lead to lipid peroxidative damage to the cell membrane [30]. The increase in MDA content can reflect the degree of lipid peroxidation and cellular damage [54]. Bile acids may exert antioxidant effects by scavenging free radicals and inhibiting oxidative stress, helping to protect cells from oxidative damage in fish [21]. Previous studies have indicated that the dietary supplementation of bile acids reduces MDA content and increases the activities of SOD and T-AOC in largemouth bass (*Micropterus salmoides*) [21], large yellow croaker [25], striped catfish (*Pangasianodon hypophthalmus*) [16], and tongue sole (*Cynoglossus semilaevis*) [55]. In agreement with these, our results showed that the MDA content in the intestine was reduced when dietary bile acids were given as high as 800 mg/kg, whereas the T-AOC level and the activity of SOD were induced in the same level of bile acid supplementation in the diets, indicating that optimal exogenous bile acid supplementation helps prevent lipid peroxidation and maintain normal oxidative/antioxidant physiological homeostasis in fish via the rapid clearance of excessive ROS [21].

## 5. Conclusions

In summary, this study demonstrates that dietary supplementation with bile acids at 800 mg/kg improves growth and increases intestinal digestive enzyme activity and antioxidant capacity, thereby protecting barrier integrity in the intestine of *L. maculatus*. Furthermore, bile acids may promote glucose metabolism in the liver by activating the FXR receptor, and they may alleviate intestinal inflammatory responses by inhibiting the transcripts of the NF-κB pathway and inflammatory cytokines.

## Figures and Tables

**Table 1:** Formulation and proximate composition of the experimental diets (g/kg dry-matter basis).

Ingredients	Bile Acids Supplementation (mg/kg Diet)			
	Con	BA400	BA800	BA1200
Fishmeal ^1^	300.00	300.00	300.00	300.00
Poultry by-product meal	80.00	80.00	80.00	80.00
Wheat gluten	30.00	30.00	30.00	30.00
Soybean meal	285.00	285.00	285.00	285.00
Wheat flour	200.00	200.00	200.00	200.00
Squid paste	10.00	10.00	10.00	10.00
Fish oil	20.00	20.00	20.00	20.00
Soybean oil	20.00	20.00	20.00	20.00
Lecithin	20.00	20.00	20.00	20.00
Ca(H_2_PO_4_)_2_	18.00	18.00	18.00	18.00
L-Ascorbate-2-phosphate	0.50	0.50	0.50	0.50
Vitamin Premix ^2^	2.00	2.00	2.00	2.00
Mineral Premix ^3^	5.00	5.00	5.00	5.00
Choline chloride	5.00	5.00	5.00	5.00
Microcrystalline cellulose	4.50	4.10	3.70	3.30
Bile acids ^4^	0.00	0.40	0.80	1.20
Proximate composition				
Crude protein	426.00	430.0	431.00	432.00
Crude lipid	121.00	118.00	119.70	117.00
Crude ash	80.40	79.80	80.40	80.30

^1^ Xiamen Jiakang feed Co., Ltd., Xiamen, China, imported from Peru (crude protein: 68.00%, crude lipid:12.00%). ^2^ Vitamin premix (mg/kg diet): Thiamin, 10 mg; Riboflavin, 8 mg; Pyridoxine HCl, 10 mg; Vitamin B12, 0.2 mg; Vitamin K3, 10 mg; Inositol, 100 mg; Pantothenic acid, 20 mg; Niacin acid, 50 mg; Folic acid, 2 mg; Biotin, 2 mg; Retinol acetate, 400 mg; Cholecalciferol, 5 mg; alpha-Tocopherol, 100 mg; Eethoxyquin, 150 mg; Wheat middling, 1.1328 g [1]. ^3^ Mineral premix (mg/kg diet): NaF, 2 mg; CuSO_4_·5H_2_O, 10 mg; KI, 0.8 mg; CoCl_2_·6H_2_O (1%), 50 mg; FeSO_4_·H_2_O, 80 mg; ZnSO_4_·H_2_O, 50 mg; MnSO_4_·H_2_O, 25 mg; MgSO_4_·7H_2_O, 200 mg; Zoelite, 4.582 g [1]. ^4^ Bile acid mixture (purity > 97%, including 75.2% hyodeoxycholic acid (HDCA), 17.4% chenodeoxycholic acid (CDCA), and 4.2% hyodeoxycholicacid (HCA)) was observed from Longchang Animal Health Co., Ltd., Dezhou, Shandong, China.

**Table 2:** Sequences of the PCR primers of *L. maculatus* used in this study.

Target Gene	Forward Primer (5′-3′)	Reverse Primer (5′-3′)	AT/°C	AE
*pepck*	CGGGAGAACATCACACACCT	CAGTGGGTCGATGATGGGAC	60	1.07
*g6pase*	CAGGTCATGGGGTACTGCTC	TTCCCGCTTTGGTTTCACCT	60	1.06
*fbp*	AACTGAGAAAGTCCCCCGAC	CCGGCCAAAACCTCGTATCT	60	0.92
*hk*	CTGGCTTGTGGGGACAGATT	GAGGCTGGCCCTCTTTATCC	60	1.05
*pk*	GTGGCCCAATCCAAATGTCC	GCAAGAGTGAGAGTTGGGGT	60	1.08
*pfk*	CGAGGGGCTAAATGTCAGGG	AAGGGGCATTCCGGTGATTT	60	0.91
*gys*	CGCATCCAGGAGTTCATCAGAGG	GTAGCGTCCAGCAATGAAGAAGAAG	60	1.09
*pygl*	TGTGATGGTTCTGTCGCTGGAG	AAGGAGTGGACGAAGATGGTGATC	60	0.98
*fxr*	GGAGGACAGGATACGCAAGAGTG	CAGGATGGTTACGGTGGTGAGG	60	0.96
*tgr5*	AGCGGTATGGTGATGGCGTAG	CATGACGGACAGCAACGACTC	60	1.03
*tnf-α*	GATCGTCATCCCACAAACCG	GCTTTGCTGCCTATGGAGTC	60	1.03
*il-1β*	GTCAACTTACGTGCACCCTG	AAATCGTACCATGTCGCTGC	60	0.95
*il-8*	GGATCAGTTTCTTCACCCAGG	CAGGTGGAGTCGAGGATCAT	60	1.00
*tgf-β*	ACCGACAATGAGCAGGGTTT	GGTGGCTGCTGATGTTTTGG	60	0.96
*il-4*	ACCATGCATTACTACAGCACTG	CACATTCAGGGGCGTTTGTC	60	1.06
*il-10*	TTCAAAACTCCGTTCGCCTG	TCACTCTTGAGCTCGTCGAA	60	0.97
*nfκb*	TGTGGTGTACGTACCGCTTC	TTCTCACACGGCTGGACTAC	60	1.03
*ikbα*	GCACGAGTGGAAGACGCAGATC	CGTCCGCCTGGTTCGTTATTACA	60	0.93
*ikkβ*	TCGGCAGCAGCTCCATCACA	AGGTGGTGCGTCTGGTGGTT	60	1.03
*ikkα*	ACAGCCAGCACCTCTTCATCCA	ACCAGCATCCAGCACGACCTT	60	1.05
*β-actin*	CAACTGGGATGACATGGAGAAG	TTGGCTTTGGGGTTCAGG	60	1.08
*18S*	GGGTCCGAAGCGTTTACT	TCACCTCTAGCGGCACAA	60	0.94

Abbreviation: AT, annealing temperature; AE, amplification efficiency; *pepck*, phosphoenolpyruvate carboxykinase; *pk*, pyruvate kinase; *pfk*, phosphofructokinase; *g6pase*, glucose-6-phosphatase; *hk*, hexokinase; *gys*, glycogen synthase; *fbp*, fructose 1,6-bisphosphatase; *pygl*, phosphorylase glycogen L; *il-8*, interleukin-8; *il-1β*, interleukin-1β; *il-4*, interleukin-4; *il-10*, interleukin-10; *tnf-α*, tumor necrosis factor-α; *tgf-β*, transforming growth factor-β; *fxr*, farnesoid X receptor; *tgr5*, trans-membrane G protein-coupled receptor-5; *nfκb*, nuclear factor *κ*b; *ikk*, inhibitor of κb kinase; *ikbα*, NF-κB inhibitory protein α; *18S*, 18S ribosomal RNA gene.

**Table 3:** The growth, feed utilization, and morphometric parameters of *L. maculatus* fed different levels of bile acids for 8 weeks.

Items	Con	BA400	BA800	BA1200
FBW (g)	130.5 ± 3.46 ^a^	135.53 ± 5.94 ^ab^	147.6 ± 2.19 ^b^	136.43 ± 6.54 ^ab^
WG (%)	539.38 ± 17.14 ^a^	562.83 ± 29.47 ^ab^	625.18 ± 10.54 ^b^	566.54 ± 31.65 ^ab^
FCR	1.22 ± 0.02	1.31 ± 0.04	1.22 ± 0.05	1.28 ± 0.08
FI (g/fish)	133.34 ± 4.8 ^a^	148.84 ± 3.76 ^b^	154.55 ± 4.35 ^b^	147.24 ± 2.33 ^b^
AFR (%)	4.41 ± 0.1	4.70 ± 0.17	4.89 ± 0.03	4.99 ± 0.40
Survival (%)	98.33 ± 1.67	95.00 ± 0.00	100.00 ± 0.00	98.33 ± 1.67

Values are mean ± SE (*n* = 3 for each treatment). Values in the same column having different superscript letters are significantly different (*p* < 0.05). Abbreviations: FBW, final body weight (g); WG, weight gain; FCR, feed conversion rate; FI, feed intake; AFR, abdominal fat ratio.

**Table 4:** The proximate composition (%, wet weight) in the whole fish of *L. maculatus* fed different levels of bile acids for 8 weeks.

Items	Con	BA400	BA800	BA1200
Moisture	69.98 ± 0.35	69.46 ± 0.41	69.15 ± 0.46	70.03 ± 0.24
Crude protein	16.7 ± 0.19	16.55 ± 0.29	16.23 ± 0.39	16.5 ± 0.08
Crude lipid	7.08 ± 0.38 ^a^	7.4 ± 0.2 ^ab^	8.15 ± 0.52 ^b^	7.3 ± 0.04 ^ab^
Crude ash	5.01 ± 0.11 ^b^	4.71 ± 0.15 ^ab^	4.57 ± 0.04 ^a^	4.75 ± 0.12 ^ab^

Values are mean ± SE (*n* = 3 for each treatment). Values in the same column having different superscript letters are significantly different (*p* < 0.05).

## Data Availability

The data that support the findings of this study are available from the corresponding author upon reasonable request.

## Supplementary Materials

The following supporting information can be downloaded at: https://www.mdpi.com/article/10.3390/ani14091299/s1, Figure S1: Effect of dietary bile acid levels on the content of the total bile acid in of *L. maculatus* reared at two temperatures for 8 weeks. References [56,57] are cited in the supplementary materials.
