# Supplementary material for: Effects of Dietary Supplementation of Bile Acids on Growth, Glucose Metabolism, and Intestinal Health of Spotted Seabass (Lateolabrax maculatus)
Source: Animals (Basel). 2024 Apr 25;14(9):1299. doi: 10.3390/ani14091299 (PMC11083208; doi:10.3390/ani14091299)
Supplement: Supplementary file 1 [file animals-14-01299-s001.zip › animals-2922964-supplementary.pdf]

Supplementary Information

Effects of dietary supplementation of bile acids on growth, glucose metabolism and intestinal health of spotted seabass (*Lateolabrax maculatus*)

Yongping Liu [1,2,#], Xiao Li [1,2,3,#], Jibin Lin [1,2,], Kai Song [1,2,], Xueshan Li [1,2,], Ling Wang [1,2,], Chunxiao Zhang [1,2,*], Kangle Lu [1,2,*]

[1] State Key Laboratory for Mariculture Breeding, Fisheries College of Jimei University, Xiamen, 361021, PR China

[2] Xiamen Key Laboratory for Feed Quality Testing and Safety Evaluation, Fisheries College of Jimei Uni-versity, Xiamen 361021, China

[3] Key Laboratory of Healthy Mariculture for the East China Sea, Ministry of Agriculture and Rural Affairs, Jimei University, Xiamen, 361021, PR China

* Corresponding authors at: Fisheries College, Jimei University, Yindou Road 43, Xiamen 361021, China.

E-mail address: cxzhang@jmu.edu.cn (C. Zhang); lukangle@jmu.edu.cn (K. Lu).

[#] Y. Liu and X. Li contributed equally to this work.

Supplementary texts

S1. Materials and methods

S1.1. Ethical statement

All procedures in this study were conducted following the guidelines of the Ethics Committee of Care and Use for Laboratory Animals of Jimei University (JMU202303004).

S1.2. Preparation of diets

In this study, the formulation and production process of the experimental diet for juvenile *L. maculatus* are consistent with that of the control group in Table 1.

S1.3. Experimental design and feeding trial

Five hundred juvenile *L. maculatus* were obtained from the commercial fish breeding farm (Zhaoan, Fujian). The fish per 250 were housed in a 1000 L semi-static system with pre-set temperatures of 27°C and 33°C, respectively. They were fed the same control diets twice daily for 2 weeks to acclimate to the experimental environment. After acclimation, 90 fish (1.09 ± 0.02 g) adapted to a water temperature of 27°C were randomly grouped into three 150 L tanks (30 fish per tank) of recirculating aquaculture systems, with a pre-set temperature of 27°C (optimal temperature). The 90 fish of the same size that acclimated to 33°C were transferred to another circulating water system consisting of 3 tanks with a pre-set temperature of 33°C. During the feeding trial for 8 weeks, fish were fed the same control diet at ~ 3% of their weight twice daily at 8:00 and 17:00. Uneaten food and feces were removed after 30 min of feeding. The dechlorinated tap water was renewed, and feed

intake was recorded daily. The conditions of the culture were as follows: water temperature at 27.0 ± 0.5°C and 33 ± 0.5°C, 12 h/12 h light/dark photoperiod, total ammonia–nitrogen < 0.34 mg/L, dissolved oxygen ≥ 6.1 mg/L, and pH 7.0 ~ 7.5.

S1.4. Sample collection

After the end of the feeding experiment, the experimental fish were starved for 24 h to purge the gut content, followed by euthanisation with eugenol (1:10000; Sigma-Aldrich, USA). For the determination of total bile acid content in the bile, 3 fish per tank were randomly selected and dissected to separate the gallbladder (n = 9). The bile was then extracted from the gallbladder using a sterile syringe, transferred to a 2-mL tube, and stored at -80°C.

S1.5 Total bile acid measurement

Total bile acid in the bile of fish were extracted and measured using the targeted metabolomics method with modifications (Hu et al., 2020; Yang et al., 2017). Briefly, bile samples were homogenized with 100 μL of acetonitrile and sonicated for 10 min at 4°C. The samples were centrifuged at 2,0000 *g* at 4°C for 15 min. The supernatants (~100 μL) were mixed with 40 μL of Na_2CO_3 solution (100 mM) and 40 μL of 2% benzoyl chloride acetonitrile solution and incubated at room temperature for 30 min. After the spike of 1.6 μL of stable-isotope labeled standards, the samples were centrifuged again at 12000 rpm for 15 min at 4°C. The supernatants (40 μL) were mixed with 10 μL of acetonitrile in 0.01% formic acid and transferred to an autosampler vial to measure the neurotransmitter concentrations on a UHPLC−MS/MS (QTrap 6500 plus, AB Sciex Co., Ltd., USA). For other quality

control/quality assurance, during the process, one blank was analyzed after five samples. The recoveries determined were 78.0−106.4% and relative standard deviations were below 10.8%.

In this study, ten bile acids, including CA (cholic acid), ACA (allocholic acid), GCDCA (glycochenodeoxycholic acid), GCA (glycocholic acid), TLCA (taurolithocholic acid), TUDCA (tauroursodeoxycholic acid), TDCA (taurodeoxycholic acid), TCDCA (taurochenodeoxycholic acid), TCA (taurocholic acid), and T-beta-MCA (tauro-β-muricholic acid), were detected in the bile of *L. maculatus*. Finally, the total sum of the content of these ten bile acids is considered as the measured total bile acid content.

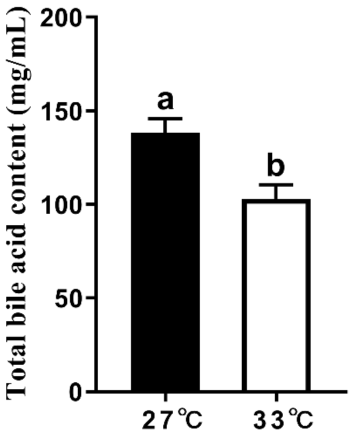

Figure S1. Effect of dietary bile acid levels on the content of the total bile acid in of *L. maculatus* reared at two temperatures for 8 weeks (n = 9). Bars with different letters are different at $p < 0.05$.

Reference:

[1] Hu, T., An, Z., Shi, C., et al., 2020. A sensitive and efficient method for simultaneous profiling of bile acids and fatty acids by UPLC-MS/MS. J Pharmaceut Biomed 178, 112815.

[2] Yang, T., Shu, T., Liu, G., et al., 2017. Quantitative profiling of 19 bile acids in rat plasma, liver, bile and different intestinal section contents to investigate bile acid homeostasis and the application of temporal variation of endogenous bile acids. J. Steroid Biochem. 172: 69-78.
